# Clinical significance of skeletal muscle density and sarcopenia in patients with pancreatic cancer undergoing first-line chemotherapy: a retrospective observational study

**DOI:** 10.1186/s12885-020-07753-w

**Published:** 2021-01-18

**Authors:** In-Ho Kim, Moon Hyung Choi, In Seok Lee, Tae Ho Hong, Myung Ah. Lee

**Affiliations:** 1 Departments of Internal Medicine, Division of Medical Oncology, Seoul St. Mary’s Hospital, The Catholic University of Korea, 222 Banpo-daero, Seocho-gu, Seoul, 137-701 South Korea; 2 Cancer Research Institute, College of Medicine, The Catholic University of Korea, Seoul, South Korea; 3 Departments of Radiology, Eunpyeong St. Mary’s Hospital, The Catholic University of Korea, Seoul, South Korea; 4 Departments of Internal Medicine, Division of Gastroenterology, Seoul St. Mary’s Hospital, The Catholic University of Korea, Seoul, South Korea; 5 Department of Hepato-Biliary-Pancreatic Cancer Center, Seoul St. Mary’s Hospital, The Catholic University of Korea College of Medicine, Seoul, South Korea; 6 Departments of Surgery, Seoul St. Mary’s Hospital, The Catholic University of Korea, Seoul, South Korea

**Keywords:** Pancreatic cancer, Chemotherapy, Prognosis, Skeletal muscle density, Sarcopenia

## Abstract

**Background:**

To investigate the clinical impact of sarcopenia and skeletal muscle density (SMD) among patients with metastatic pancreatic adenocarcinoma who underwent palliative first line gemcitabine-based chemotherapy.

**Methods:**

A total of 330 patients treated with first line gemcitabine-based chemotherapy between January 2010 and March 2017 were included. CT scans before chemotherapy and after 8±2 weeks were evaluated. The L3 skeletal muscle index (SMI) was used to detect sarcopenia and calculated as the total area of the L3 skeletal muscle divided by the height-squared (cm2/m2). SMD was quantified as the mean muscle radiation attenuation of the muscle cross-sectional area across the L3 vertebral body level and was assessed between − 29 and + 150 Hounsfield units.

**Results:**

A SMI to SMD comparison revealed a positive correlation (*R*2 = 0.058, *P* < 0.001). Compared with high SMD, the risks of low SMI were 1.516 (95% confidence interval [CI]: 1.164–1.973) among patients with low SMD. Kaplan–Meier analysis showed that the low SMD was related to poor overall survival (OS, median, 6.1 versus [vs.] 7.9 months, *P* = 0.010). Multivariate analysis using Cox regression showed that low SMI (hazard ratio [HR]: 1.35, 95% CI: 1.03–1.78, *P* = 0.032) and low SMD (HR: 1.45, 95% CI: 1.09–1.93, *P* = 0.011) were poor prognostic factors for OS, respectively. Co-presence of low SMI and low SMD had more powerful prognostic implication for OS (HR: 1.58, 95% CI: 1.12–2.23, *P* = 0.010). Grade 3 or higher toxicity of chemotherapy was more frequently observed in patients who have a low SMI (43% vs. 59%, *P* = 0.019) and low SMD (44% vs. 60%, *P* = 0.023). OS was not related to SMD status among patients who were chemotherapy responders (complete or partial responses). However, among non-responders (stable or progressive disease), low SMD groups had significantly poorer OS in comparison with high SMD groups (median, 5.6 vs 7.4 months, *P* = 0.006).

**Conclusions:**

Sarcopenia and SMD status can be considered a prognostic factor in patients with metastatic pancreatic adenocarcinoma who received palliative first line gemcitabine-based chemotherapy. Severe chemotherapy toxicity occurred in the sarcopenia and low SMD groups. Our data suggest that a comprehensive assessment of skeletal muscle parameters may be more useful prognostic factors.

**Supplementary Information:**

The online version contains supplementary material available at 10.1186/s12885-020-07753-w.

## Background

Metastatic pancreatic adenocarcinoma (mPCa) is one of the most aggressive types of cancer [1]. Although systemic chemotherapy with agents such as gemcitabine plus nab-paclitaxel and FOLFIRINOX show clinical benefits, mPCa has a dismal prognosis with a median overall survival (OS) of < 1 year [2, 3]. Cancer cachexia, experienced by most patients with mPCa, is related to poor prognosis, which has warranted numerous studies on the factors that affect it [4–6]. This is important for patients who receive chemotherapy because, although chemotherapy can give survival benefits to patients, it causes toxicity and can lead to physical inactivity. In this regard, sarcopenia is related to morbidity, mortality, and a decreased quality of life [7–9]. Sarcopenia is also related to chemotherapy-induced toxicity [10].

Several studies have recently been conducted on the relationship between the cancer prognosis and the patient’s skeletal muscle density (SMD) [11, 12]. SMD is a radiological characteristic and a low SMD reflects intramuscular adipose tissue infiltration and poor ‘quality’ skeletal muscle, which is related to poor muscle strength [13].

The application of computed tomography (CT) in clinical practice has led to improvement of skeletal muscle parameter evaluation and is considered the gold standard for studying such parameters [14]. In this study, we aimed to investigate the clinical impact of sarcopenia and SMD by CT scan among patients with mPCa who undergo palliative first line gemcitabine-based chemotherapy.

## Methods

### Study population

A total of 330 patients with mPCa treated with palliative first line gemcitabine-based chemotherapy between January 2010 and March 2017 were initially included in this study. Among them, 79 patients who either did not undergo baseline CT scans within 2 weeks of the initiation of chemotherapy, had Eastern Cooperative Oncology Group performance status scores of 3–4, or were experiencing other systemic medical problems such as infection were excluded, resulting in 251 patients in the study. We conducted baseline CT scans before chemotherapy and after 8±2 weeks to evaluate chemotherapy responses. All diagnoses were confirmed via biopsy or aspiration of the primary tumor or metastatic lesion. The chemotherapy regimens consisted of gemcitabine alone or in combination with other agents. Radiological changes were evaluated using the Response Evaluation Criteria in Solid Tumors version 1.1 [15]. Objective response was defined as complete response (CR) or partial response (PR), while disease control was defined as CR, PR, or stable disease (SD).

### Body composition assessment

CT images acquired before the chemotherapy were retrieved for analysis. One axial portal phase image was selected at the level of the third lumbar vertebral body transverse process. Skeletal muscle area measurement was conducted on the selected axial image by using a commercially available system (Advantage Windows workstation 4.6, GE Healthcare, Milwaukee, Wisconsin, USA). Skin, visceral organs, and the central spinal canal were excluded manually when drawing the area containing the abdominal wall and back muscles on the axial image. The areas of the abdominal wall and back muscles were calculated based on the areas of the pixels with attenuation between − 29 and 150 Hounsfield units (HU) in the demarcated areas.

The L3 skeletal muscle index (SMI) was used to detect sarcopenia and was calculated as the total area of the L3 skeletal muscle divided by the height-squared (cm^2^/m^2^). The cut-off points for SMI were defined as 43 and 53 cm^2^/m^2^ for non-overweight (body mass index [BMI]< 25 kg/m^2^) and overweight men (BMI≥25 kg/m^2^), respectively, and as 41 cm^2^/m^2^ for women [16]. SMD was quantified as the mean muscle radiation attenuation (in HU) of the muscle cross-sectional area across the L3 vertebral body level and was assessed between − 29 and + 150 HU [17]. The cut-off points for SMD were set at 41 and 33 cm^2^/m^2^ for non-overweight and overweight patients, respectively [16]. Total fat area was calculated by the sum of visceral and subcutaneous adipose tissue.

### Endpoint of study

The primary endpoint is to evaluate the survival according to SMI and SMD. The secondary endpoint is to evaluate the chemotherapy response and toxicities according to SMI and SMD.

### Statistical analysis

The correlation between clinicopathologic factors and both SMI and SMD were analysed using the Pearson’s chi-square test and linear-by-linear association. The correlation between SMI and SMD was determined using the Pearson’s chi-square test and t-test. The correlation between chemotherapy response and both SMI and SMD were analysed using the Pearson’s chi-square test. OS and progression-free survival (PFS) were calculated from the start date of first-line palliative chemotherapy until the date of death from any cause or of disease progression, respectively. For survival analyses, living patients or those with no disease progression were censored from the last follow-up date. Univariate analyses of OS and PFS were performed using the Kaplan-Meier method and log-rank test. Multivariate Cox regression forward models with significant potential risk factors (*p*-value < 0.2 in univariate analysis) were used to verify the prognostic values of SMI, SMD and co-presence of SMI and SMD, and were individually adjusted for age, sex, performance status, tumor site, histology, number of metastatic organs, CA19–9 level, and chemotherapy regimen. All analyses were performed using SPSS software (version 24; IBM Corp., Armonk, NY), and a two-sided *P* < 0.05 was considered statistically significant.

### Ethics

The study was performed according to the Helsinki declaration and approved by the Institutional Review Board of Seoul St. Mary’s Hospital.

## Results

### Baseline patient characteristics

**Fig. 1:**
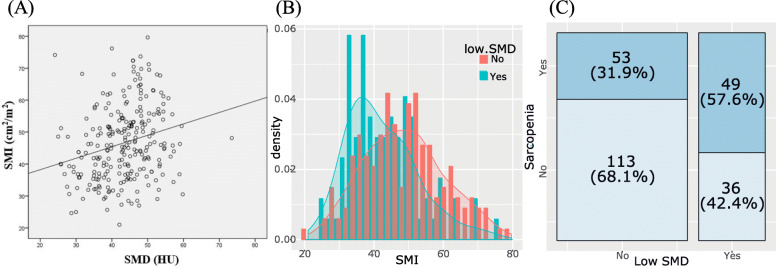
**a** Correlation between skeletal muscle index (SMI) and skeletal muscle density (SMD) **b** SMI according to SMD status **c** Relation between SMD status and sarcopenia

A total of 251 patients were included in the analysis; their characteristics are listed in Table 1. Low SMD was related to high total fat areas. Female was associated with low SMI. The correlation between SMI and SMD was assessed (Fig. 1). A positive correlation between SMI and SMD was found (*R*^2^ = 0.058, *P* < 0.001). The low-SMD group exhibited a lower SMI than the high-SMD group (42.31±10.18 vs. 48.77±11.67 cm^2^/m^2^, *P* < 0.001). Among the 166 patients with high SMD before chemotherapy, 53 (31.9%) had low SMI, whereas among the 85 patients with low SMD before chemotherapy, 49 (57.6%) had low SMI. Compared with high SMD, the risks of low SMI were 1.516 (95% confidence interval [CI]: 1.164–1.973) among patients with low SMD. Moreover, 49 of all 251 patients (19.5%) had both low SMI and low SMD.

**Table 1:** Baseline characteristics

	All patients	SMI			SMD		
		High (*n* = 149, 59%)	Low (*n* = 102, 41%)	*P*	High (*n* = 166, 66%)	Low (*n* = 85, 34%)	*P*
**Age, median (range)**	63.4 ± 9.4	64.5 ± 9.1	61.7 ± 9.7	0.019	63.8 ± 9.0	62.6 ± 10.2	0.336
< 65	137 (54.6%)	69 (46.3%)	68 (66.7%)	0.002	88 (53.0%)	49 (57.6%)	0.573
≥65	114 (45.4%)	80 (53.7%)	34 (33.3%)		78 (47.0%)	36 (42.4%)	
**Sex**				< 0.001			0.582
Female	90 (35.9%)	70 (47.0%)	20 (19.6%)		62 (37.3%)	28 (32.9%)	
Male	161 (64.1%)	79 (53.0%)	82 (80.4%)		104 (62.7%)	57 (67.1%)	
**ECOG**				0.802			0.368
0	26 (10.4%)	17 (11.4%)	9 8.8%)		16 (9.6%)	10 (11.8%)	
1	182 (72.5%)	107 (71.8%)	75 (73.5%)		125 (75.3%)	57 (67.1%)	
2	43 (17.1%)	25 (16.8%)	18 (17.6%)		25 (15.1%)	18 (21.2%)	
**Location**				0.524			0.980
Head	129 (51.4%)	81 (54.4%)	48 (47.1%)		85 (51.2%)	44 (51.8%)	
Body	52 (20.7%)	29 (19.5%)	23 (22.5%)		35 (21.1%)	17 (20.0%)	
Tail	70 (27.9%)	39 (26.2%)	31 (30.4%)		46 (27.7%)	24 (28.2%)	
**Histological type**				0.319			0.300
Well diff.	24 (9.6%)	14 (9.4%)	10 (9.8%)		18 (10.8%)	6 (7.1%)	
Moderate diff.	151 (60.2%)	95 (63.8%)	56 (54.9%)		104 (62.7%)	47 (55.3%)	
Poor diff.	41 (16.3%)	24 (16.1%)	17 (16.7%)		24 (14.5%)	17 (20.0%)	
Unknown	35 (13.9%)	16 (10.7%)	19 (18.6%)		20 (12.0%)	15 (17.6%)	
**Number of metastatic organs**				0.991			0.615
Only one (1)	134 (53.4%)	79 (53.0%)	55 (53.9%)		91 (54.8%)	43 (50.6%)	
More than one (≥2)	117 (46.6%)	70 (47.0%)	47 (46.1%)		75 (45.2%)	42 (49.4%)	
**CA19–9**	7216.2 ± 22900.6	5097.8 ± 15422.6	10310.7 ± 30549.6	0.114	6390.7 ± 21320.2	8828.4 ± 25767.8	0.454
				0.512			0.034
Normal	48 (19.1%)	31 (20.8%)	17 (16.7%)		25 (15.1%)	23 (27.1%)	
Elevated	203 (80.9%)	118 (79.2%)	85 (83.3%)		141 (84.9%)	62 (72.9%)	
**First line chemotherapy**				0.889			0.930
Gemcitabine single	91 (36.3%)	53 (35.6%)	38 (37.3%)		61 (36.7%)	30 (35.3%)	
Gemcitabine based chemotherapy	160 (63.7%)	96 (64.4%)	64 (62.7%)		105 (63.3%)	55 (64.7%)	
**BMI**	21.7 ± 3.1	21.8 ± 3.1	21.6 ± 3.0	0.666	21.8 ± 3.4	21.6 ± 2.5	0.514
**Total fat area**	43.3 ± 7.8	144.6 ± 96.3	157.0 ± 85.3	0.298	125.8 ± 76.7	196.3± 101.6	< 0.001
**SMA**	122.1 ± 26.9	137.9 ± 20.6	98.9 ± 15.8	< 0.001	127.2 ± 26.6	112.1 ± 24.6	< 0.001
**SMI**	46.6 ± 11.6	54.0 ± 8.5	35.7 ± 4.9	< 0.001	48.8 ± 11.7	42.3 ± 10.2	< 0.001
**SMD**	43.3 ± 7.8	45.1 ± 7.4	40.7 ± 7.8	< 0.001	47.7 ± 5.2	34.8 ± 4.4	< 0.001

*BMI* body mass index, *CA19–9* carbohydrate antigen 19–9, *diff* differentiation, *ECOG* Eastern Cooperative Oncology Group, *SMA* skeletal muscle area, *SMD* skeletal muscle density, *SMI* skeletal muscle index

### Survival according to SMI and SMD

**Fig. 2:**
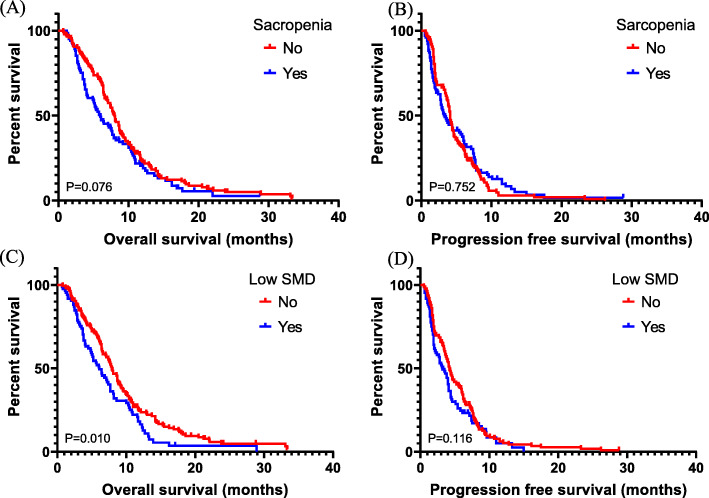
Survival according to SMI and SMD. SMI status was not associated with OS (median, 6.0 versus 8.0 months in patients with low and high SMI, respectively; *P* = 0.076) (**a**) or with PFS (median, 3.3 versus 4.1 months in patients with low and high SMI, respectively; *P* = 0.752) (**b**). A low SMD was associated with poorer OS than a high SMD (6.1 vs 7.9 months, *P* = 0.010) (**c**), but not with a poorer PFS (3.2 vs 4.2 months, *P* = 0.116) (**d**)

**Fig. 3:**
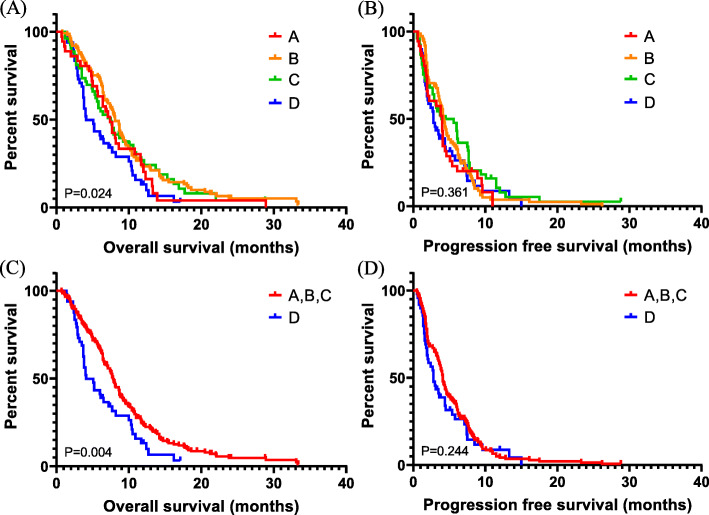
Survival of patients with both low SMI (sarcopenia) and low SMD compared to other patients. Group A: High SMI and high SMD; Group B: High SMI and low SMD; Group C: Low SMI and high SMD; Group D: Low SMI and low SMD. Patients with low SMI and low SMD showed poorer overall survival than those with either of these conditions or neither one (**a**, **c**). Copresence of low SMI and low SMD were not associated with progression-free survival (**b**, **d**)

OS and PFS were assessed according to SMI and SMD (Fig. 2). With the univariate analysis, low SMI was not related to OS (median, 6.0 versus 8.0 months; *P* = 0.076) or PFS (*P* = 0.752). Patients with low SMD had poorer OS than those with high SMD (6.1 versus 7.9 months, *P* = 0.010). However, there were no differences in PFS (*P* = 0.116) with respect to SMD. Furthermore, patients with both low SMI and low SMD showed poorer OS than others (4.1 vs 7.8 months, *P* = 0.004) (Fig. 3). We also performed a multivariate Cox proportional hazard regression for SMI, SMD, and baseline characteristics (Table 2). Low SMI, low SMD, and co-presence of low SMI and low SMD were statistically significant prognostic factors for OS but not for PFS (Low SMI, hazard ratio [HR]: 1.35, 95% confidence interval [CI]: 1.03–1.78, *P* = 0.032; low SMD, HR: 1.45, 95% CI: 1.09–1.93, *P* = 0.011; and Co-presence of low SMI and low SMD, HR: 1.58, 95% CI: 1.12–2.23, *P* = 0.010). Based on the multivariate analysis results, the C index of low SMI, low SMD, and co-presence of low SMI and low SMD were 0.64, 0.65 and 0.66, respectively. We evaluated the relation between change pattern (increase or decrease) of SMI or SMD and survival during chemotherapy. There is no OS and PFS difference according to change pattern of SMI or SMD. Moreover, Eastern Cooperative Oncology Group performance status and type of first line chemotherapy regimen were prognostic factors for OS, while the number of metastatic sites and baseline CA19–9 levels were related to both OS and PFS (Supplementary Material 1).

**Table 2:** Multivariate analysis for SMI and SMD with Cox regression for overall survival

		HR	95% CI		*P*
SMI	High	1			
	Low	1.352	1.03	1.78	0.032
SMD	High	1			
	Low	1.451	1.09	1.93	0.011
Low SMI and low SMD	No	1			
	Yes	1.579	1.12	2.23	0.010

*CI* confidence interval, *HR* hazard ratio, *OS* overall survival, *SMD* skeletal muscle density

SMI, SMD, and co-presence of low SMI and low SMD were individually adjusted for age, sex, ECOG, location, histological type, number of metastatic organs, CA19–9, first line chemotherapy

### Chemotherapy response and toxicities according to SMI and SMD

Chemotherapy response was assessed according to SMI, SMD, and their rate of change (Table 3). Objective responses were not related to low SMI or low SMD (mean SMI: CR/PR vs. SD/progressive disease [PD], 46.58 vs. 46.62 cm^2^/m^2^, *P* = 0.981; mean SMD: CR/PR vs. SD/PD, 43.27 vs. 43.48, respectively, HU, *P* = 0.856). Disease control was also not related to low SMI or low SMD (mean SMI: CR/PR/SD vs. PD, 47.21 vs. 45.44 cm2/m2, *P* = 0.249; mean SMD: CR/PR/SD vs. PD,43.58 vs. 42.85, respectively, HU, *P* = 0.484). We also assessed the correlation between chemotherapy response and the rate of change of SMI and SMD and found that objective response was not related to change in SMI or SMD (SMI change: CR/PR vs. SD/PD, − 1.99 vs. -4.15%, respectively, *P* = 0.157; SMD change: CR/PR vs. SD/PD, -2.88 vs. -1.10%, respectively, *P* = 0.298). Disease control was also not related to change (SMI change: CR/PR/SD vs. PD, -3.95 vs. -3.05%, respectively, *P* = 0.298; SMD change (%), CR/PR/SD vs, PD, − 1.67 vs. -1.26, respectively, *P* = 0.784).

**Table 3:** Correlation between chemotherapy response and SMI, SMD, and their rate of change

	CR/PR (*n* = 60)	SD/PD (*n* = 191)	*P*	CR/PR/SD (*n* = 163)	PD (*n* = 88)	*P*
SMI	46.58 ± 11.46	46.62 ±12.07	0.981	47.21±11.94	45.44±10.88	0.249
SMI change (%)	−1.99 ± 11.46	−4.15±9.74	0.157	−3.95 ± 10.59	−3.05±9.77	0.501
SMD	43.27±7.95	43.48±7.52	0.856	43.58±8.22	42.85±7.09	0.484
SMD change (%)	−2.88 ± 11.45	−1.10±11.48	0.298	−1.67 ± 11.11	−1.26±12.17	0.784

*CR* complete response, *PD* progressive disease, *PR* partial response, *SD* stable disease, *SMD* skeletal muscle density, *SMI* skeletal muscle index. Values are in Hounsfield units

We also investigated the relationship between chemotherapy-related toxicities and SMI or SMD (Table 4). Low SMI and low SMD were not related to grade 3 or higher neutropenia, anaemia, thrombocytopenia, fatigue, or diarrhea, separately. However, all grade 3 or higher adverse events were more frequently reported by patients with low SMI (43% by high-SMI patients vs. 59% by low-SMI patients, *P* = 0.019) as well as by patients with low SMD (44% by high-SMD patients vs. 60% by low-SMD patients, *P* = 0.023).

**Table 4:** Association between toxicity and skeletal muscle parameters

	SMI			SMD		
	High (*n* = 149, 59%)	Low (*n* = 102, 41%)	*P*	High (*n* = 166, 66%)	Low (*n* = 85, 34%)	*P*
all grade ≥3 toxicity	64 (43%)	60 (59%)	0.019	73 (44%)	51 (60%)	0.023
neutropenia	32 (22%)	28 (28%)	0.348	35 (21%)	25 (29%)	0.191
anemia	21 (14%)	21 (21%)	0.237	24 (15%)	18 (21%)	0.242
thrombocytopenia	14 (9%)	15 (15%)	0.275	18 (11%)	11 (13%)	0.777
fatigue	18 (12%)	21 (21%)	0.100	24 (15%)	15 (18%)	0.634
diarrhea	2 (1%)	1 (1%)	0.999	2 (1%)	1 (1%)	0.999

*SMD* skeletal muscle density, *SMI* skeletal muscle index

### Survival rates among chemotherapy responders and non-responders

**Fig. 4:**
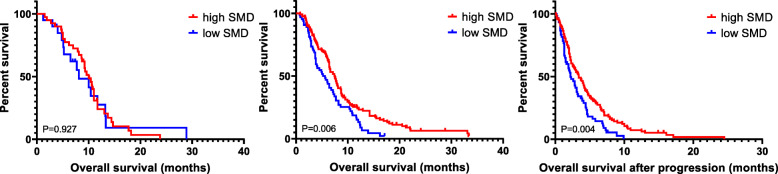
Survival according to SMD among chemotherapy responders vs. non-responders. Among responders (CR/PR), OS was not associated with SMD status (**a**). Among non-responders (SD/PD), patients with low SMD showed poorer OS than those with high SMD (median, 5.6 vs. 7.4 months, *P* = 0.006) (**b**). Among non-responder, patients with low SMD also showed poorer survival after progression at 8 weeks of initiation of chemotherapy than their high SMD-group counterparts (2.2 vs. 3.4 months, *P* = 0.004) (**c**)

We assessed survival according to the presence of low SMI and low SMD in both chemotherapy responders and non-responders (Fig. 4). OS was not associated with SMD status among responders (CR/PR); however, among non-responder patients (SD/PD), the low SMD group showed poorer OS than the high SMD group (median: 5.6 vs 7.4 months, *P* = 0.006). Thus, we analysed survival after progression at 8 weeks of initiation of chemotherapy according to SMD status among non-responders. In this case, the low SMD group showed poorer survival after progression at 8 weeks than the high SMD group (median: 2.2 vs. 3.4 months, *P* = 0.004). Moreover, OS was not associated with SMI in either responder (*P* = 0.489) or non-responder patients (*P* = 0.061).

## Discussion

In this study, we assessed the clinical impact of sarcopenia and SMD status in patients with mPCa who received palliative first line gemcitabine-based chemotherapy. To our knowledge, our study on the prognostic value of both sarcopenia and SMD status is the first study to evaluate comprehensively the association between skeletal muscle parameters, SMI and SMD with the largest cohort of patients with metastatic pancreatic cancer.

Several previous studies have shown how sarcopenia and low SMD are a negative prognostic factors for patients with cancer, which overlaps with our finding [4, 18]. Sarcopenia is the age-related decline of muscle mass and function/strength [19]. Several factors have been indicated to play a role in the onset and progression of sarcopenia. Meng, S. J et al. suggested that oxidative stress and molecular inflammation play important roles in age-related muscle atrophy [20]. SMD is reported as the mean radiation attenuation in Hounsfield Units on CT imaging [21]. SMD can vary greatly, and muscle with lower SMD has increased fat production or infiltration [13]. Both SMD and SMI have been shown to predict survival in patients with various types of cancer [12, 16].

Our results showed that SMD is positively correlated with SMI. Several previous studies showed that SMI and SMD are positively correlated, in accordance with our research [21, 22]. However, other studies showed no significant correlation between SMI and SMD [23, 24]. It is likely that the cutoff values differed from one study to another and there might be differences among the study populations in terms of cancer type, stage of disease, sex, and age. Our study is particular consisted of those at advanced disease stages (stage 4) and, therefore, with very poor prognoses. In general, decreases in SMD are detected earlier than decreases in SMI. Furthermore, CT-based calculations allow for early detection of decreases in HUs (SMD), even when the muscle area remains unchanged [25]. Because our patients were at an advanced stage with poor prognoses, it is likely that they experienced decreases in both SMD and skeletal muscle area (SMA), which could result in the significant correlation observed between SMI and SMD. This suggests that, when assessing a patient using skeletal muscle parameters as prognostic factors, it may be useful to simultaneously evaluate SMI and SMD, rather than just one of these parameters.

Our results showed that SMD was a better prognostic factor than SMI in terms of statistical significance. Some previous studies also showed that low SMD is a better prognostic factor than SMI [12, 25–28]. Similar to our findings, three previous studies [12, 25, 28] found that a low SMD was significantly associated with poor OS while sarcopenia was not, suggesting that SMD is a more reliable prognostic factor than sarcopenia status. This may also explain findings from a previous study that showed low SMD leads to muscle weakness independently of muscle area, resulting in higher prognostic value [17]. Additionally, intermuscular fat development reflects the level of physical activity [29, 30] and has also been associated with severe inflammation [31], suggesting that these patients are more likely to encounter a higher number of severe adverse events during chemotherapy. It is also possible that SMD could be a more accurate measurement of muscle function and, therefore, precedes the development of sarcopenia development [26]. Also, our results showed that analysis using both SMI and SMD is of better prognostic value than SMI or SMD alone in terms of statistical significance, which has clinical applicability. Our data suggest that comprehensive assessment of skeletal muscle parameters may be more useful prognostic factors.

Based on these results, we also determined whether the prognostic role of the skeletal muscle parameters is associated with the chemotherapy effects. Neither sarcopenia status nor SMD had any association with chemotherapy response. This result supports that sarcopenia or low SMD is related to OS, but not PFS. Furthermore, changes in these parameters were not related to chemotherapy responses. These results are inconsistent with the results from previous studies. For instance, Chu, M et al. showed that high SMD was strongly associated with radiographic complete responses [21]; however, Daly et al. showed no correlation between skeletal muscle parameters and chemotherapy response [32]. In fact, comparisons with previous studies may not be feasible because variables such as the type of cancer, purpose of chemotherapy, and chemotherapy regimens differed among studies. If no association between chemotherapy response and skeletal muscle parameters is determined, it may be due to the relatively low baseline level of the skeletal muscle parameter, as well as and the rate of change for which no statistical significance could be found. Our results suggested that sarcopenia or low SMD is a prognostic factor, but it is not clear whether sarcopenia or low SMD is a predictive marker for chemotherapy. Further studies are needed to clarify this issue.

Our data revealed that severe chemotherapy toxicity was associated with low SMI and low SMD, which was consistent with previous studies [7, 24]. This could be due to the link between body composition and drug pharmacokinetics and has important clinical implications. Mir, O., et al. suggested other hypotheses that systemic inflammation underlies sarcopenia, and might play a role in the occurrence of toxicities [33]. Patients with sarcopenia or low SMD should be considered for prevention and aggressive management of chemotherapy toxicity.

Although no survival rate differences were observed according to SMD in patients who responded to chemotherapy, non-responder patients with low SMD showed poor survival time after disease progression at 8 weeks. In other words, the worse the chemotherapy response, the better SMD works as a prognostic factor. This should be taken into account when deciding whether to perform second line chemotherapy or best supportive care only after disease progression. As clinicians consider several factors, such as performance status, when deciding whether to provide additional chemotherapy, weakness of skeletal muscle may also be helpful in the decision-making process.

There are limitations to our study owing to its retrospective nature. The first is that the skeletal muscle parameters were evaluated using CT, which represents a single aspect of the muscle functional status. It would be optimal to assess muscle depletion from the perspectives of both function and strength; which should be considered in future studies. The second limitation is that other parameters that reflect nutrition and health status such as food intake, and albumin and C-reactive protein levels were not investigated.

## Conclusion

Our results showed that SMD and sarcopenia could be considered as prognostic factors in patients with mPCa who received palliative first line gemcitabine-based chemotherapy. Severe toxicity of chemotherapy occurred in the sarcopenia and low SMD groups. Our data suggest that comprehensive assessment of skeletal muscle parameters may be useful prognostic factors.

## Supplementary Information

**Additional file 1.** Multivariate analysis for OS and PFS with Cox regression

## Data Availability

The data that support the findings of this study are available from the corresponding author but restrictions apply to the availability of these data, which were used under license for the current study, and so are not publicly available. Data are however available from the corresponding author upon reasonable request and with permission of Institutional Review Board of the Seoul St. Mary’s Hospital.

## Abbreviations

BMI: Body mass index
CI: Confidence interval
CR: Complete response
CT: Computed tomography
HR: Hazard ratio
HU: Hounsfield units
mPCa: metastatic pancreatic adenocarcinoma
OS: Overall survival
PD: Progressive disease
PFS: Progression-free survival
PR: Partial response
SD: Stable disease
SMA: Skeletal muscle area
SMD: Skeletal muscle density
