# Supplementary material for: Clinical significance of skeletal muscle density and sarcopenia in patients with pancreatic cancer undergoing first-line chemotherapy: a retrospective observational study
Source: BMC Cancer. 2021 Jan 18;21:77. doi: 10.1186/s12885-020-07753-w (PMC7814715; doi:10.1186/s12885-020-07753-w)
Supplement: Supplementary file 1 — Additional file 1. Multivariate analysis for OS and PFS with Cox regression [file 12885_2020_7753_MOESM1_ESM.docx]

Supplementary Material 1. Multivariate analysis for OS and PFS with Cox regression.

|  | OS | | | |  | PFS | | | |  | OS | | | |  | PFS | | | |  | OS | | | |  | PFS | | | |
| --- | --- | --- | --- | --- | --- | --- | --- | --- | --- | --- | --- | --- | --- | --- | --- | --- | --- | --- | --- | --- | --- | --- | --- | --- | --- | --- | --- | --- | --- |
|  | HR | 95% CI | | P |  | HR | 95% CI | | P |  | HR | 95% CI | | P |  | HR | 95% CI | | P |  | HR | 95% CI | | P |  | HR | 95% CI | | P |
| **ECOG** |  |  |  |  |  |  |  |  |  |  |  |  |  |  |  |  |  |  |  |  |  |  |  |  |  |  |  |  |  |
| 0 | 1 |  |  |  |  |  |  |  |  |  | 1 |  |  |  |  |  |  |  |  |  | 1 |  |  |  |  |  |  |  |  |
| 1 | 1.78 | 1.09 | 2.89 | 0.02 |  |  |  |  |  |  | 1.86 | 1.15 | 3.02 | 0.01 |  |  |  |  |  |  | 1.86 | 1.15 | 3.03 | 0.01 |  |  |  |  |  |
| 2 | 2.87 | 1.63 | 5.04 | <0.001 |  |  |  |  |  |  | 2.89 | 1.64 | 5.08 | <0.001 |  |  |  |  |  |  | 2.75 | 1.56 | 4.85 | <0.001 |  |  |  |  |  |
| **Number of metastatic organs** |  |  |  |  |  |  |  |  |  |  |  |  |  |  |  |  |  |  |  |  |  |  |  |  |  |  |  |  |  |
| Only one (1) | 1 |  |  |  |  | 1 |  |  |  |  | 1 |  |  |  |  | 1 |  |  |  |  | 1 |  |  |  |  | 1 |  |  |  |
| More than one (≥2) | 1.68 | 1.28 | 2.20 | <0.001 |  | 1.50 | 1.14 | 1.97 | 0.004 |  | 1.58 | 1.21 | 2.07 | 0.001 |  | 1.50 | 1.14 | 1.97 | 0.004 |  | 1.65 | 1.26 | 2.16 | <0.001 |  | 1.50 | 1.14 | 1.97 | 0.004 |
| **CA19-9 elevation** |  |  |  |  |  |  |  |  |  |  |  |  |  |  |  |  |  |  |  |  |  |  |  |  |  |  |  |  |  |
| No | 1 |  |  |  |  | 1 |  |  |  |  | 1 |  |  |  |  | 1 |  |  |  |  | 1 |  |  |  |  | 1 |  |  |  |
| Yes | 1.54 | 1.08 | 2.20 | 0.018 |  | 1.52 | 1.08 | 2.14 | 0.017 |  | 1.64 | 1.14 | 2.35 | 0.007 |  | 1.52 | 1.08 | 2.14 | 0.017 |  | 1.51 | 1.06 | 2.16 | 0.024 |  | 1.52 | 1.08 | 2.14 | 0.017 |
| **First line chemotherapy** |  |  |  |  |  |  |  |  |  |  |  |  |  |  |  |  |  |  |  |  |  |  |  |  |  |  |  |  |  |
| Gemcitabine single | 1 |  |  |  |  |  |  |  |  |  | 1 |  |  |  |  |  |  |  |  |  | 1 |  |  |  |  |  |  |  |  |
| Gmecitabine-based combination | 0.69 | 0.52 | 0.91 | 0.008 |  |  |  |  |  |  | 0.70 | 0.53 | 0.93 | 0.014 |  |  |  |  |  |  | 0.68 | 0.51 | 0.90 | 0.007 |  |  |  |  |  |
| **SMI** |  |  |  |  |  |  |  |  |  |  |  |  |  |  |  |  |  |  |  |  |  |  |  |  |  |  |  |  |  |
| High | 1 |  |  |  |  |  |  |  |  |  |  |  |  |  |  |  |  |  |  |  |  |  |  |  |  |  |  |  |  |
| Low | 1.35 | 1.03 | 1.78 | 0.032 |  |  |  |  |  |  |  |  |  |  |  |  |  |  |  |  |  |  |  |  |  |  |  |  |  |
| **SMD** |  |  |  |  |  |  |  |  |  |  |  |  |  |  |  |  |  |  |  |  |  |  |  |  |  |  |  |  |  |
| High |  |  |  |  |  |  |  |  |  |  | 1 |  |  |  |  |  |  |  |  |  |  |  |  |  |  |  |  |  |  |
| Low |  |  |  |  |  |  |  |  |  |  | 1.45 | 1.09 | 1.93 | 0.011 |  |  |  |  |  |  |  |  |  |  |  |  |  |  |  |
| **Low SMI and low SMD** |  |  |  |  |  |  |  |  |  |  |  |  |  |  |  |  |  |  |  |  |  |  |  |  |  |  |  |  |  |
| No |  |  |  |  |  |  |  |  |  |  |  |  |  |  |  |  |  |  |  |  | 1 |  |  |  |  |  |  |  |  |
| Yes |  |  |  |  |  |  |  |  |  |  |  |  |  |  |  |  |  |  |  |  | 1.58 | 1.12 | 2.23 | 0.010 |  |  |  |  |  |

CA19-9, carbohydrate antigen 19-9; CI, confidence interval; ECOG, Eastern Cooperative Oncology Group; HR, hazard ratio; OS, overall survival; PFS, progression survival; SMD, skeletal muscle density; SMI, skeletal muscle index.
